# Health-related Quality of Life in Localized and Metastatic Renal Cell Carcinoma: Insights from Patient-reported Outcome Measures

**DOI:** 10.1016/j.euros.2025.12.017

**Published:** 2026-01-21

**Authors:** Matthijs Duijn, Hilin Yildirim, Corina van den Hurk, Arnoud W. Postema, Maureen J.B. Aarts, Katja Aben, Martijn G.H. van Oijen, Adriaan D. Bins, Patricia J. Zondervan

**Affiliations:** a Department of Urology, Amsterdam UMC, University of Amsterdam, Amsterdam, The Netherlands; b Department of Research and Development, Netherlands Comprehensive Cancer Organisation, Utrecht, The Netherlands; c Department of Medical Oncology, Cancer Center Amsterdam, Amsterdam UMC, University of Amsterdam, Amsterdam, The Netherlands; d Catharina Hospital, Eindhoven, The Netherlands; e Santeon, Utrecht, The Netherlands; f Department of Urology, Leiden University Medical Center, Leiden, The Netherlands; g Department of Medical Oncology, GROW School for Oncology and Reproduction, Maastricht University Medical Centre+, Maastricht, The Netherlands; h Department of Research and Development, Netherlands Comprehensive Cancer Organisation, Utrecht, The Netherlands; i Science Department of IQ Health, Radboud University Medical Centre, Nijmegen, The Netherlands

**Keywords:** Renal cell carcinoma, Localized, Metastatic, Health-related quality of life, Patient-reported outcome measures

## Abstract

**Background and objective:**

Patient-reported outcome measures (PROMs) are increasingly being used to evaluate health-related quality of life (HRQoL) in cancer care. We investigated the effects of treatment for both localized (M0) and metastatic renal cell carcinoma (mRCC) on generic and cancer-specific HRQoL up to 15 wk after diagnosis.

**Methods:**

Patients were selected from the National PROspective infrastructure for renal cell carcinoma (PRO-RCC), including M0 and mRCC patients who participated in the HRQoL assessment. HRQoL was measured using the Dutch validated European Organization for Research and Treatment of Cancer Quality of Life Questionnaire-Core 30 at baseline (T_0_), 15 wk (T_1_), and 6 mo (T_2_) after diagnosis. Within-group HRQoL changes over time were analyzed using paired t tests for patients with data available at multiple time points (T_0_ vs T_1_). Exploratory analyses comparing T_0_ versus T_2_ were also performed.

**Key findings and limitations:**

A total of 295 patients were included, of whom 217 (73.6%) had M0 disease and 78 (26.4%) had mRCC. At follow-up, 89 patients with M0 disease (41%) and 38 with mRCC (48.7%) completed the T_1_ assessment. In the M0 group, significant improvements at T_1_ were observed in scores for emotional functioning (80.2 vs 87.4; change [Δ] 7.2, 95% confidence interval [CI] 3.8–10.5; *p* < 0.001), social functioning (81.8 vs 86.7; Δ 4.9, 95% CI 0.2–9.6, *p* = 0.042), insomnia (31.5 vs 22.9; Δ −8.6, 95% CI −13.9 to −3.4; *p* = 0.002), and appetite loss (11.6 vs 6.4; Δ −5.2, 95% CI −9.3 to −1.2; *p* = 0.012). In the mRCC group, significant improvements at T_1_ were noted in scores for emotional functioning (77.9 vs 84.7; Δ 6.8, 95% CI −1.4 to 14.9; *p* = 0.015), fatigue (31.6 vs 23.7; Δ −7.9, 95% CI −15.5 to −0.3; *p* = 0.042), and pain (18.4 vs 7.5; Δ −10.9, 95% CI −19.8 to −2.1; *p* = 0.017).

**Conclusions and clinical implications:**

Patients with M0 disease and mRCC experienced improvements in several functional and symptom domains within 15 wk after diagnosis. Future studies should assess the impact of these changes on treatment continuation and/or responses.

**Patient summary:**

We looked at health-related quality of life (HRQoL) in patients with localized or metastatic kidney cancer. In general, both groups reported improvements in emotional, social, and physical wellbeing at 15 weeks after their diagnosis, and results suggest further changes at 6 months after diagnosis. Patients reported improvements in symptoms such as fatigue, pain, sleep, and appetite. Our results indicate that patients can find meaningful improvements in their quality of life shortly after diagnosis and initiation of treatment.

## Take Home Message

Early and ongoing quality-of-life monitoring is crucial for patients with renal cell carcinoma. Improvements in emotional, social, and physical wellbeing are observed at 15 wk after diagnosis, with exploratory analyses suggesting potential further changes 6 mo after diagnosis. These findings highlight the need for tailored support and continued research beyond the early postdiagnosis period.

## 1 Introduction

Renal cell carcinoma (RCC) accounts for approximately 80% of all kidney cancer diagnoses [1], [2]. The overall 5-yr survival rate is 74%, but rates differ by disease stage at diagnosis: 93% for localized RCC (M0) versus 17% for metastatic RCC (mRCC) [3]. Key risk factors include lifestyle behaviors (eg, smoking, lack of physical activity), underlying health conditions (eg, hypertension, chronic kidney disease), environmental exposures, and genetic mutations such as loss of the *VHL* gene [4], [5]. Treatment strategies are primarily based on disease stage and risk assessment and take into account the histological RCC subtype, as subtypes differ in their genetic, biological, and clinical characteristics. According to international treatment guidelines, M0 RCC is typically managed with partial nephrectomy (PN) or radical nephrectomy, with alternative treatment modalities such as thermal ablation, stereotactic ablative body radiotherapy, or active surveillance for patients who are not fit for surgery. For high-risk disease, adjuvant pembrolizumab is recommended for clear-cell RCC after surgery [6]. For mRCC, systemic therapies, including immuno-oncology agents and tyrosine kinase inhibitors, are the mainstay of treatment [7]. However, these treatments, which often involve multiple mechanisms of action, induce side effects and prolonged use can lead to greater toxicity and delayed onset of side effects. These adverse effects may necessitate treatment discontinuation or dose adjustments, which can negatively impact the overall treatment efficacy [8], [9]. Furthermore, the patient’s health-related quality of life (HRQoL) may be compromised.

HRQoL refines the broad concept of QoL by focusing on aspects directly related to a person’s health that are likely to be influenced by health care interventions. Most HRQoL definitions include dimensions of physical, mental, and social functioning, alongside overall wellbeing and perceptions of general health [10]. HRQoL evaluation is an essential component when assessing the impact of treatments or illnesses on an individual’s overall wellbeing. The significance of HRQoL assessment has grown, particularly as patient perspectives and experiences related to side effects are increasingly influencing shared decision-making processes [11]. Patient-reported outcomes (PROs), which are health-related data directly provided by patients without external interpretation or modification, represent the gold standard for HRQoL assessment. PRO measures (PROMs) consist of standardized, validated questionnaires completed by patients in the periods before, during, and/or after treatment period. These instruments address generic, disease-specific, and treatment-related issues and yield valuable insights for health care professionals and patients. To date, real-world studies evaluating HRQoL in patients with RCC remain limited, despite the recognized importance of HRQoL in informing clinical decision-making for both health care providers and patients. Furthermore, RCC-specific PROMs are seldom used as standalone instruments and are typically administered alongside generic or cancer-specific PROMs [12], [13]. The PROMs most commonly used in RCC include the Functional Assessment of Cancer Therapy-Kidney Symptom Index (FKSI) [14], European Organization for Research and Treatment of Cancer Quality of Life Questionnaire-Core 30 (EORTC QLQ-C30) [15], Functional Assessment of Cancer Therapy-General (FACT-G) [16], and EuroQol 5-Dimension Visual Analog Scale [17]. Motzer et al [18] also identified numerous symptom-specific PROMs. Owing to the limited availability of PRO data in RCC and the lack of commonly used PROMs in this population, the aim of our study was to evaluate generic and cancer-specific HRQoL in patients with M0 or mRCC from baseline to 15 wk after diagnosis. We also conducted exploratory analyses to assess HRQoL outcomes at 6 mo after diagnosis. Our hypothesis was that patients with M0 or mRCC experience significant improvements in HRQoL within 15 wk after diagnosis [19], [20], [21], [22].

## 2 Patients and methods

### 2.1 Patient selection

Patients were identified from the national PRO-RCC database, a multicenter prospective registry for all RCC patients in the Netherlands. This observational cohort is structured for continuous enrollment and longitudinal monitoring of individuals newly diagnosed with M0 or mRCC [23]. Patients aged ≥18 yr with clinical suspicion for or histopathologically proven (m)RCC and signed informed consent are eligible for inclusion. All patients diagnosed with M0 or mRCC who participated in the HRQoL assessment part of PRO-RCC were included. Patients who did not complete the baseline questionnaire before treatment initiation (*n* = 128) were excluded. The study was approved by the medical ethics committees of all hospitals participating in PRO-RCC.

### 2.2 HRQoL

A PROM assessing HRQoL was administered at diagnosis/baseline (T_0_), 15 wk (T_1_), and 6 mo (T_2_). The PROM was the Dutch validated version of the EORTC QLQ-C30 [15]. This tool is designed to systematically evaluate and provide comprehensive insights into the HRQoL of cancer patients. The QLQ-C30 questionnaire assesses various scales and domains related to an individual’s health status [15]. Supplementary Table 1 lists the QLQ-C30 domains, scales, and reference values [24]. Higher scores on the global health status/QoL and functional scales correspond to better HRQoL, whereas lower scores on the symptom scales reflect fewer symptoms and better HRQoL. The scores for each scale were calculated using the formulas outlined in the EORTC QLQ-C30 scoring manual [15].

### 2.3 Statistical analysis

All statistical analyses were conducted using SPSS v28.0. The normality of continuous variables was evaluated via visual inspection, consistent with the prespecified statistical analysis plan, which deemed the paired t test appropriate given its robustness to minor deviations from normality. The homogeneity of variance was assessed using Levene’s test. Within-group changes in HRQoL over time (T_0_ vs T_1_) were examined using paired t tests, and 95% confidence intervals (CIs) for the mean change (Δ) were calculated. Exploratory analyses comparing T_0_ versus T_2_ scores were also performed. Missing data were managed using complete-case analyses for each comparison.

## 3 Results

Between January 2023 and March 2025, a total of 295 patients were enrolled in the study. Follow-up questionnaires were completed by 89 patients with M0 disease, corresponding to a dropout rate of 59%. Among patients with mRCC, 38 individuals completed the T_1_ questionnaires, corresponding to a dropout rate of 49%. Baseline characteristics of the full cohort are summarized in Table 1.

**Table 1:** Baseline characteristics of the study cohort by metastasis status

Parameter	No metastasis (M0)	Metastatic RCC
Patients, *n* (%)	217 (73)	78 (27)
Mean age, yr (standard deviation)	64.6 (10.6)	67.9 (9.4)
Male, *n* (%)	152 (70)	62 (80)
Mean body mass index, kg/m^2^ (standard deviation)	27.4 (4.6)	29.2 (16.3)
Comorbidity, *n* (%)^a^		
Heart disease	45 (21)	12 (15)
Stroke	4 (1.8)	3 (3.9)
Hypertension	100 (46)	33 (42)
Asthma, chronic bronchitis, COPD	30 (14)	10 (13)
Diabetes	36 (17)	12 (15)
Kidney disease	115 (53)	39 (50)
Liver disease	6 (2.8)	0 (0)
Anemia/other blood disease	19 (8.8)	11 (14)
Thyroid disease	10 (4.6)	0 (0)
Depression	6 (2.8)	3 (3.9)
Arthrosis	52 (24)	16 (21)
Backache	71 (33)	27 (35)
Rheumatism	9 (4.2)	1 (1.3)
Hereditary cancer syndrome	5 (2.3)	0 (0)
Treatment, *n* (%)		
Surgical	142 (65)	0 (0)
Partial nephrectomy	41 (29)	0 (0)
Radical nephrectomy	49 (35)	0 (0)
Not specified	52 (36)	0 (0)
Ablative therapy	13 (6.0)	0 (0)
Radiotherapy	3 (1.4)	0 (0)
Systemic therapy	0 (0)	30 (39)
IO^b^	0 (0)	29 (97)
TKI	0 (0)	1 (3.3)
Surgery + IO	7 (3.2)	2 (2.6)
Radiotherapy + IO	0 (0)	1 (1.3)
Active surveillance	22 (10)	0 (0)
Best supportive care/watchful waiting	0 (0)	9 (12)
Other	7 (3.2)	2 (2.6)
Unknown	43 (20)	34 (44)

COPD = chronic obstructive pulmonary disease; IO = immuno-oncology agent; RCC = renal cell carcinoma; TKI = tyrosine kinase inhibitor.

a PRO.

b Including IO monotherapy and IO + IO or IO + TKI combinations.

### 3.1 QLQ-C30 scores

**Fig. 1:**
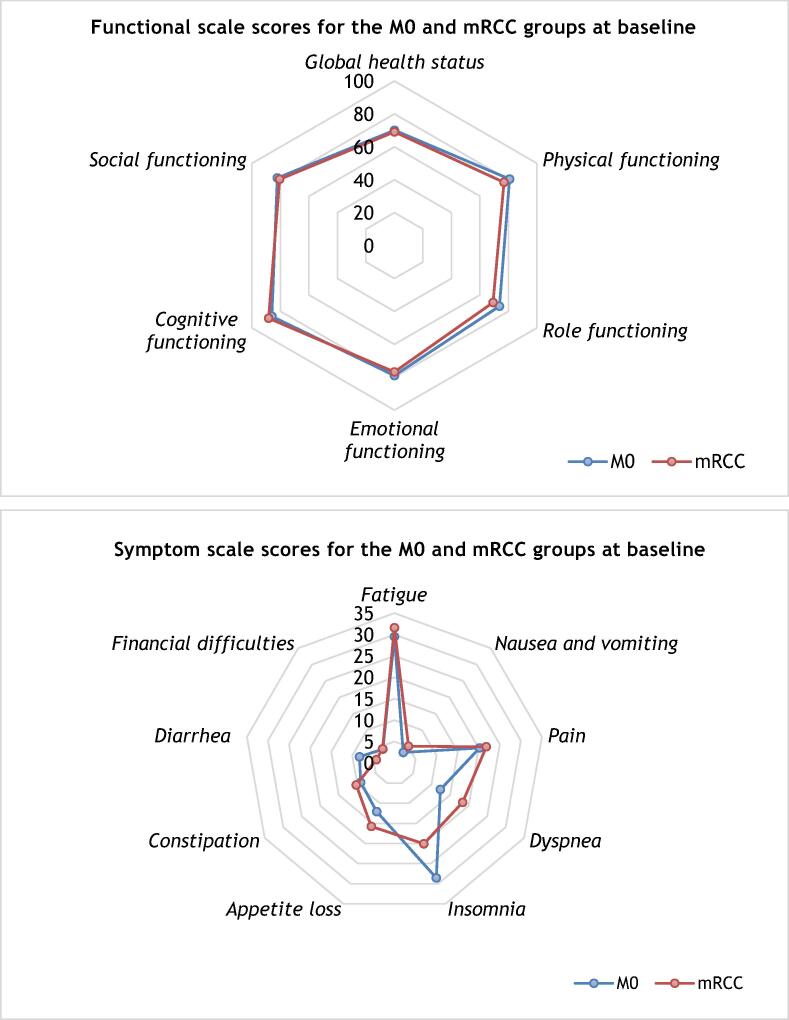
QLQ-C30 scores for the M0 (*n* = 217) and mRCC (*n* = 78) groups at baseline. mRCC = metastatic renal cell carcinoma.

Baseline QLQ-C30 scores for the M0 and mRCC groups are presented in Fig. 1 and Supplementary Table 2.

#### 3.1.1 Scores in the M0 group at T_0_ and T_1_

**Fig. 2:**
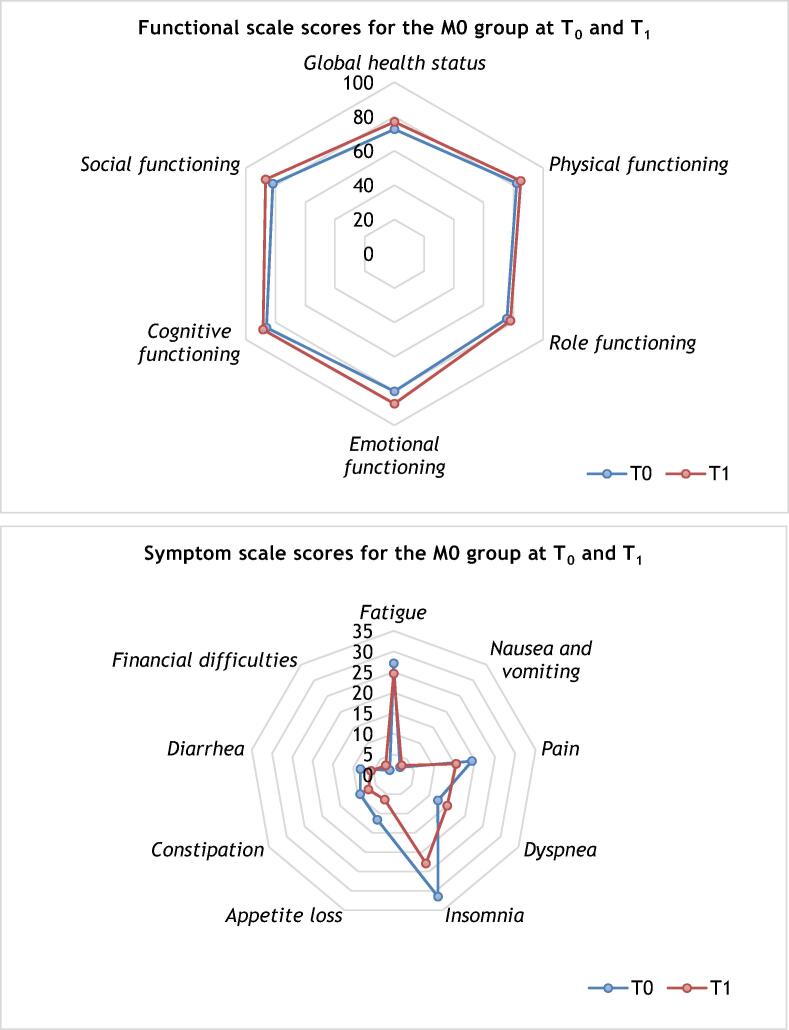
QLQ-C30 scores for the M0 group at times T_0_ and T_1_ (*n* = 89).

Fig. 2 shows QLQ-C30 scores in the M0 group at T_0_ and T_1_. At T_1_, patients experienced statistically significant improvements in mean scores for global health status (72.6 vs 76.9; Δ 4.3, 95% CI 0.4–8.2; *p* = 0.03), emotional function (80.2 vs 87.4; Δ 7.2, 95% CI 3.8–10.5; *p* < 0.001), and social functioning (81.8 vs 86.7; Δ 4.9, 95% CI 0.2–9.6; *p* = 0.042) in comparison to T_0_. There were also significant improvements at T_1_ in insomnia (31.5 vs 22.9; Δ −8.6, 95% CI −13.9 to −3.4; *p* = 0.002) and appetite loss (11.6 vs 6.4; Δ −5.2, 95% CI −9.3 to −1.2; *p* = 0.012; Supplementary Table 3).

#### 3.1.2 Scores in the mRCC group at T_0_ and T_1_

**Fig. 3:**
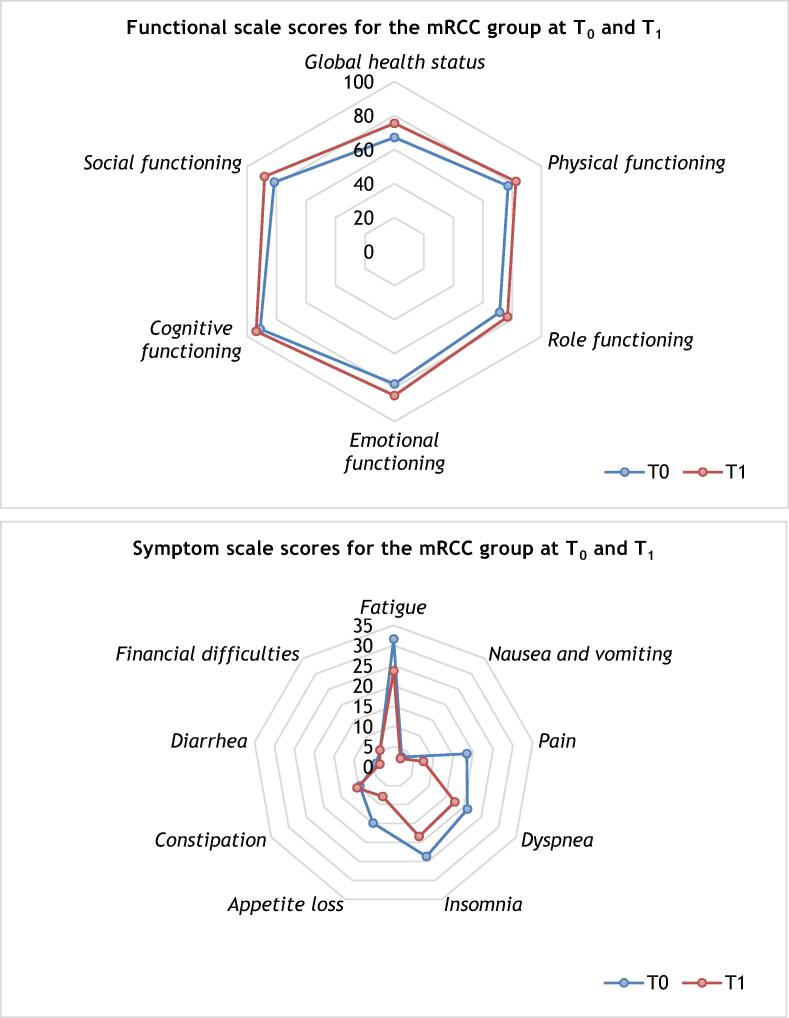
QLQ-C30 scores for the mRCC group at times T_0_ and T_1_ (*n* = 38). mRCC = metastatic renal cell carcinoma.

In the mRCC group, improvements in mean scores for global health status (67.1 vs 75.4; Δ 8.3, 95% CI 0.4–16.3; *p* = 0.040) and emotional functioning (77.9 vs 84.7; Δ 6.8, 95% CI −12.2 to 1.4; *p* = 0.015) were observed at T_1_ in comparison to T_0_. At T_1_, patients reported less fatigue (31.6 vs 23.7; Δ −7.9, 95% CI −15.5 to −0.3; *p* = 0.042) and pain (18.4 vs 7.5; Δ −10.9, 95% CI −19.8 to −2.1; *p* = 0.017) in comparison to T_0_ (Fig. 3 and Supplementary Table 4).

#### 3.1.3 Changes in scores from T_0_ to T_2_ in the M0 and mRCC groups

Results for the exploratory analyses of changes in scores from T_0_ to T_2_ are provided in Supplementary Tables 5 and 6.

## 4 Discussion

The aim of our study was to evaluate the generic and cancer-specific HRQoL of patients with M0 or mRCC at diagnosis and up to 15 wk after diagnosis. Exploratory analyses also assessed HRQoL at 6 mo after diagnosis. Descriptive analysis revealed that baseline QLQ-C30 scores, with the exception of the financial domain, were consistently lower in our RCC cohort than in the general Dutch population (Supplementary Table 7) [25]. The data for the Dutch general population were collected in March–April 2017 via an online international cross-sectional HRQoL survey. Participants were recruited in equal numbers across ten sex- and age-based strata to ensure that the sample was representative of the internet-using general population.

During follow-up, patients in our study cohort reported improvements across multiple functional scales. However, these observations should be interpreted with caution, as the T_1_ measurements represent the same cohort assessed at baseline, albeit with fewer participants owing to loss to follow-up. No formal analyses were conducted to evaluate differences by treatment type, and the inclusion of patients under active surveillance or watchful waiting at baseline further limits our ability to attribute the improvements observed to specific treatments. In the overall M0 and mRCC cohort, scores for some specific symptom scales, including nausea and vomiting, dyspnea, constipation, and financial difficulties, showed no statistically significant difference over time, although temporary deterioration was observed. These temporary fluctuations may reflect ongoing treatment effects or recovery processes, but causal inferences cannot be drawn. Overall, the functional changes observed may relate to adaptation to disease and treatment or greater clarity regarding prognosis, but mechanistic explanations require specifically designed analyses and cannot be inferred from the current data [26].

In the group with M0 disease, improvements at T_1_ were observed in scores for emotional functioning, social functioning, insomnia, and appetite loss. However, the examination of multiple outcomes increases the probability of obtaining statistically significant results by chance; therefore, these findings should be interpreted with caution. Among patients with mRCC, scores for emotional functioning, fatigue, and pain improved at T_1_. Similarly, given the number of outcomes analyzed, the statistical significance of individual results should be interpreted with caution. Exploratory analyses of long-term outcomes (Supplementary Tables 5 and 6) should likewise be viewed with caution owing to the high attrition rate at T_2_.

To facilitate interpretation of the statistically significant mean changes in scores between baseline and T_1_ across multiple functional and symptom scales, the changes were evaluated against established thresholds for clinical meaningfulness, as presented in Supplementary Table 1. At baseline, mean scores for functional and symptom scales showing significant improvement were, on average, above (for functional scales) or below (for symptom scales) the clinically relevant reference values defined by Giesinger et al. [24]. However, the wide standard deviations suggest considerable interpatient variability, and not all patients necessarily exceeded these thresholds.

Although additional improvements were observed at a subsequent time point, the magnitude and statistical significance of these changes varied across subgroups and domains; however, their clinical relevance remains uncertain in the absence of validated thresholds.

To the best of our knowledge, this is one of the first real-world studies to evaluate HRQoL in patients with RCC using PROMs. Sidoti Abate et al. [27] conducted a comprehensive HRQoL analysis within the ROBOCOP II trial comparing robot-assisted PN (RAPN) to open PN in patients with M0 RCC. RAPN was associated with lower pain scores across multiple assessments up to postoperative day 30, indicating short-term analgesic benefits. Patients undergoing RAPN also reported modest improvements in gastrointestinal symptoms, general symptoms, and fatigue at discharge. However, by postoperative day 90, there were no significant differences in any domain assessed between the groups.

The CheckMate 214 trial of nivolumab + ipilimumab versus sunitinib assessed HRQoL in previously untreated patients with advanced or mRCC. The trial demonstrated significant improvements in HRQoL favoring nivolumab + ipilimumab in four of the five FKSI-19 domains (disease-related symptoms, physical disease-related symptoms, treatment side effects, and functional wellbeing), as well as in the physical and functional wellbeing domains of the FACT-G [20]. These results are partly consistent with our findings. Previous studies have also investigated HRQoL in patients with other urological malignancies, such as bladder cancer. For instance, Nuijens et al. [18] evaluated the impact of treatment for localized muscle-invasive bladder cancer during the first 2 yr after diagnosis. In contrast to that study, we refrain from describing our findings as “sustained”, as our design did not permit longitudinal within-patient assessment across all time points. Another study on HRQoL after bladder cancer did not identify significant differences in functional domains or symptom scores across treatment modalities or disease stages [28].

HRQoL research in kidney cancer remains methodologically challenging and inconsistent. Gross et al. [13] conducted a systematic review of PROM use in RCC and found that nearly half of the studies included did not use any RCC-specific PROMs to assess HRQOL, which raises concerns about the relevance and sensitivity of the instruments used. This lack of disease-specific assessment tools limits the ability to capture meaningful patient-centered outcomes and hinders the interpretability and clinical utility of study findings. Bergerot et al. [29] concluded that current HRQoL measures for RCC require substantial refinement and called for the development of a dedicated RCC-specific HRQoL instrument. Ultimately, the use of validated, disease-specific HRQoL measures that address domains relevant to RCC patients will be crucial for generating data that can effectively answer predefined research questions and guide patient-centered care.

To integrate RCC-specific PROMs into routine clinical practice, several strategies can be implemented. PROM administration at diagnosis would provide a personalized baseline by capturing RCC-specific symptoms, functional limitations, and psychosocial concerns before treatment initiation. During systemic therapy, regular PROM assessments (eg, at each clinic visit or treatment cycle) could facilitate monitoring of the symptom burden and treatment tolerability, and thus timely supportive care interventions and shared decision-making [11]. Incorporation of these PROMs into electronic health record systems would allow automated calculation of scores and facilitate efficient clinical interpretation. Post-treatment PROM results could identify persistent HRQoL issues, guide referrals to supportive care services, and inform individualized follow-up schedules that are based on patient needs.

As previously mentioned, this is one of the first prospective, population-based cohort studies on RCC-specific HRQoL, and included baseline (before treatment) assessments and multiple measurements of HRQoL over time (15 wk and 6 mo after diagnosis). In addition, HRQoL was assessed in M0 and mRCC groups.

Nonetheless, our study has several limitations. First, the questionnaire response rate at T_1_ (43.1%) was relatively low, which limits the overall sample size and precludes meaningful subgroup analyses. Although T_1_ nonresponders did not significantly differ from T_1_ responders in terms of disease stage or physical functioning, they reported higher mean T_0_ scores for financial difficulties (nonresponders 5.3 vs responders 2.7) and slightly lower mean T_0_ scores for global health status (nonresponders 68 vs responders 70.7; Supplementary Table 8). These results suggest that our study population represents a somewhat healthier and more socioeconomically advantaged subset of the cohort who completed the HRQoL questionnaires at T_1_, with potential for overestimation of HRQoL outcomes. This pattern is consistent with known response bias in QoL research, whereby individuals with a greater symptom burden or poorer health may be less likely to participate and complete questionnaires. Furthermore, attrition over time—potentially because of disease progression or mortality—may have led to bias and further underestimation of the negative impact on HRQoL, particularly at later time points. In addition, the follow-up period, limited to a maximum of 6 mo after diagnosis, is insufficient for adequate evaluation of the impact of systemic therapy on HRQoL in patients with mRCC. The small number of patients with mRCC at T_1_ (*n* = 38) limits the robustness of the findings in this subgroup, and subtle but clinically meaningful changes in HRQoL among these patients may have gone undetected. Future research should aim to address this gap. Finally, our study was observational in nature and should therefore be regarded as hypothesis-generating rather than confirmatory. Consequently, the findings do not establish causal relationships and should be interpreted with caution pending validation in future confirmatory studies.

## 5 Conclusions

Our findings indicate that patients with M0 disease or mRCC experience measurable improvements across multiple functional and symptom domains within the first 15 wk following diagnosis. These early changes demonstrate that HRQoL is a dynamic construct and emphasize the need for systematic and ongoing assessment in clinical practice to detect clinically relevant shifts that may warrant timely supportive or therapeutic interventions. The results further support the integration of RCC-specific PROMs for more accurate capture of disease-related experiences in this population. Future research using larger, more representative cohorts and extended follow-up should determine how longitudinal HRQoL trajectories relate to treatment continuation and responses, and whether incorporation of such information into routine care can meaningfully inform clinical decision-making and improve patient outcomes.

  ***Author contributions***: Matthijs Duijn had full access to all the data in the study and takes responsibility for the integrity of the data and the accuracy of the data analysis.

  *Study concept and design*: Duijn, Yildirim, Zondervan.

*Acquisition of data*: Duijn, Yildirim, Aben.

*Analysis and interpretation of data*: Duijn, Yildirim.

*Drafting of the manuscript*: Duijn, Yildirim.

*Critical revision of the manuscript for important intellectual content*: Zondervan, van den Hurk, Postema, Aarts, van Oijen, Bins.

*Statistical analysis*: Duijn, Yildirim.

*Obtaining funding*: None.

*Administrative, technical, or material support*: Zondervan, van den Hurk, Postema, Aarts, Aben, van Oijen, Bins.

*Supervision*: Zondervan.

*Other*: None.

  ***Financial disclosures:*** Matthijs Duijn certifies that all conflicts of interest, including specific financial interests and relationships and affiliations relevant to the subject matter or materials discussed in the manuscript (eg, employment/affiliation, grants or funding, consultancies, honoraria, stock ownership or options, expert testimony, royalties, or patents filed, received, or pending), are the following: None.

  ***Funding/Support and role of the sponsor*:** None.

  ***Acknowledgments*:** We would like to thank the data managers of the Netherlands Cancer Registry for data collection.

## References

[1] Linehan W.M., Rathmell W.K. (2012). Kidney cancer. Urol Oncol.

[2] Escudier B., Porta C., Schmidinger M. (2019). Renal cell carcinoma: ESMO clinical practice guidelines for diagnosis, treatment and follow-up. Ann Oncol.

[3] National Cancer Institute. Surveillance, Epidemiology, and End Results program. SEER*Explorer. https://seer.cancer.gov/statistics-network/explorer/.

[4] Scélo G., Larose T.L. (2018). Epidemiology and risk factors for kidney cancer. J Clin Oncol.

[5] Jonasch E., Walker C.L., Rathmell W.K. (2020). Clear cell renal cell carcinoma ontogeny and mechanisms of lethality. Nat Rev Nephrol.

[6] Powles T., Tomczak P., Park S.H. (2022). Pembrolizumab versus placebo as post-nephrectomy adjuvant therapy for clear cell renal cell carcinoma (KEYNOTE-564): 30-month follow-up analysis of a multicentre, randomised, double-blind, placebo-controlled, phase 3 trial. Lancet Oncol.

[7] Ljungberg B., Bex A., Albiges L. (2024).

[8] Ellithi M., Elnair R., Chang G.V. (2020). Toxicities of immune checkpoint inhibitors: itis-ending adverse reactions and more. Cureus.

[9] Clark P.E., Schover L.R., Uzzo R.G. (2001). Quality of life and psychological adaptation after surgical treatment for localized renal cell carcinoma: impact of the amount of remaining renal tissue. Urology.

[10] Apolone G., De Carli G., Brunetti M. (2001). Health-related quality of life (HR-QOL) and regulatory issues. An assessment of the European Agency for the Evaluation of Medicinal Products (EMEA) recommendations on the use of HR-QOL measures in drug approval. Pharmacoeconomics.

[11] Gratzke C., Seitz M., Bayrle F. (2009). Quality of life and perioperative outcomes after retroperitoneoscopic radical nephrectomy (RN), open RN and nephron-sparing surgery in patients with renal cell carcinoma. BJU Int.

[12] Kluzek S., Dean B., Wartolowska K.A. (2022). Patient-reported outcome measures (PROMs) as proof of treatment efficacy. BMJ Evid Based Med.

[13] Gross F., Rasmussen I.M.L., Beisland E.G. (2025). Health-related quality of life assessment in renal cell cancer: a scoping review. Eur Urol Oncol.

[14] Rosenblad A.K., Sundqvist P., Westman B., Ljungberg B. (2021). A psychometric evaluation of the Functional Assessment of Cancer Therapy-Kidney Symptom Index (FKSI-19) among renal cell carcinoma patients suggesting an alternative two-factor structure. Qual Life Res.

[15] Aaronson N.K., Ahmedzai S., Bergman B. (1993). The European Organization for Research and Treatment of Cancer QLQ-C30: a quality-of-life instrument for use in international clinical trials in oncology. J Natl Cancer Inst.

[16] Cella D., Tulsky D.S., Gray G. (1993). The Functional Assessment of Cancer Therapy scale: development and validation of the general measure. J Clin Oncol.

[17] Herdman M., Gudex C., Lloyd A. (2011). Development and preliminary testing of the new five-level version of EQ-5D (EQ-5D-5L). Qual Life Res.

[18] Motzer R.J., Rane P.P., Saretsky T.L. (2023). Patient-reported outcome measurement and reporting for patients with advanced renal cell carcinoma: a systematic literature review. Eur Urol.

[19] Cella D., Escudier B., Tannir N.M. (2018). Quality of life outcomes for cabozantinib versus everolimus in patients with metastatic renal cell carcinoma: METEOR phase III randomized trial. J Clin Oncol.

[20] Cella D., Grünwald V., Escudier B. (2019). Patient-reported outcomes of patients with advanced renal cell carcinoma treated with nivolumab plus ipilimumab versus sunitinib (CheckMate 214): a randomised, phase 3 trial. Lancet Oncol.

[21] Motzer R.J., Powles T., Atkins M.B. (2022). Final overall survival and molecular analysis in IMmotion151, a phase 3 trial comparing atezolizumab plus bevacizumab vs sunitinib in patients with previously untreated metastatic renal cell carcinoma. JAMA Oncol.

[22] Nuijens S.T., van Hoogstraten L.M.C., Terpstra N.B. (2025). The impact of treatment for muscle-invasive bladder cancer on health-related quality of life. BJU Int.

[23] Yildirim H., Widdershoven C.V., Aarts M.J. (2023). The PRO-RCC study: a long-term prospective renal cell carcinoma cohort in the Netherlands, providing an infrastructure for ‘trial within cohorts’ study designs. BMC Cancer.

[24] Giesinger J.M., Loth F.L.C., Aaronson N.K. (2020). Thresholds for clinical importance were established to improve interpretation of the EORTC QLQ-C30 in clinical practice and research. J Clin Epidemiol.

[25] de Ligt K.M., Aaronson N.K., Liegl G. (2023). Updated normative data for the EORTC QLQ-C30 in the general Dutch population by age and sex: a cross-sectional panel research study. Qual Life Res.

[26] Secinti E., Tometich D.B., Johns S.A. (2019). The relationship between acceptance of cancer and distress: a meta-analytic review. Clin Psychol Rev.

[27] Sidoti Abate M.A., Menold H.S., Neuberger M. (2024). Quality-of-life outcomes of the ROBOtic-assisted versus conventional open partial nephrectomy (ROBOCOP) II trial. BJU Int.

[28] Catto J.W.F., Downing A., Mason S. (2021). Quality of life after bladder cancer: a cross-sectional survey of patient-reported outcomes. Eur Urol.

[29] Bergerot C.D., Mercier B.D., Castra D.V. (2023). Health-related quality of life (HR-QOL) measures in renal cell carcinoma (RCC): patient-reported relevance of items of the FKSI-19, EORTC QLQ-C30, and EQ-5D. J Clin Oncol.

