# Supplementary material for: Health-related Quality of Life in Localized and Metastatic Renal Cell Carcinoma: Insights from Patient-reported Outcome Measures
Source: Eur Urol Open Sci. 2026 Jan 21;84:50–7. doi: 10.1016/j.euros.2025.12.017 (PMC12859803; doi:10.1016/j.euros.2025.12.017)
Supplement: Supplementary Data 1 [file mmc1.docx]

**Supplementary Table 1**. EORTC QLQ-C30 thresholds for clinical importance (24).

| *Scales* | *Domains* | *Reference values* |
| --- | --- | --- |
| Functional scales | Physical functioning | >83 |
|  | Role functioning | >58 |
|  | Emotional functioning | >71 |
|  | Cognitive functioning | >75 |
|  | Social functioning | >58 |
| Symptom scales/items | Fatigue | ≤39 |
|  | Nausea and vomiting | ≤8 |
|  | Pain | ≤25 |
|  | Dyspnea | ≤17 |
|  | Insomnia | ≤50 |
|  | Appetite loss | ≤50 |
|  | Constipation | ≤50 |
|  | Diarrhoea | ≤17 |
|  | Financial difficulties | ≤17 |
