# Supplementary material for: Health-related Quality of Life in Localized and Metastatic Renal Cell Carcinoma: Insights from Patient-reported Outcome Measures
Source: Eur Urol Open Sci. 2026 Jan 21;84:50–7. doi: 10.1016/j.euros.2025.12.017 (PMC12859803; doi:10.1016/j.euros.2025.12.017)
Supplement: Supplementary Data 2 [file mmc2.docx]

**Supplementary Table 2**. QLQ-C30 scores for M0 and mRCC at baseline.

|  | QLQ-C30 scores M0 (n= 217), mean (SD) | QLQ-C30 scores mRCC (n= 78), mean (SD) |
| --- | --- | --- |
| Global health status/QoL |  |  |
| *Global health status/QoL* | 70.1 (20.7) | 69.1 (23.8) |
| Functional scales |  |  |
| *Physical functioning* | 80.8 (20.7) | 76.9 (20.2) |
| *Role functioning* | 73.7 (31.1) | 69.2 (29.7) |
| *Emotional functioning* | 79.0 (19.5) | 76.8 (21.2) |
| *Cognitive functioning* | 86.0 (18.2) | 88.3 (17.6) |
| *Social functioning* | 82.2 (23.1) | 80.6 (25.4) |
| Symptom scales/items |  |  |
| *Fatigue* | 29.5 (27.1) | 31.6 (27.5) |
| *Nausea and vomiting* | 3.2 (10.1) | 5.1 (12.1) |
| *Pain* | 20.2 (26.6) | 21.8 (26.5) |
| *Dyspnea* | 12.4 (20.9) | 18.4 (24.4) |
| *Insomnia* | 28.6 (31.8) | 20.1 (26) |
| *Appetite loss* | 12.1 (21.5) | 15.8 (21.6) |
| *Constipation* | 9.1 (20.9) | 10.3 (21) |
| *Diarrhoea* | 8.3 (19.6) | 4.3 (12.4) |
| *Financial difficulties* | 4.2 (15.3) | 4.3 (12.4) |

*SD standard deviation.*
