# Supplementary material for: Health-related Quality of Life in Localized and Metastatic Renal Cell Carcinoma: Insights from Patient-reported Outcome Measures
Source: Eur Urol Open Sci. 2026 Jan 21;84:50–7. doi: 10.1016/j.euros.2025.12.017 (PMC12859803; doi:10.1016/j.euros.2025.12.017)
Supplement: Supplementary Data 3 [file mmc3.docx]

**Supplementary Table 3**. QLQ-C30 scores for M0 at T0 and T1.

|  | QLQ-C30 scores T0 (n=89), mean (SD) | QLQ-C30 scores T1 (n=89), mean (SD) | Δ QLQ-C30 scores (95% C.I.) | *p* |
| --- | --- | --- | --- | --- |
| Global health status/QoL^1^ |  |  |  |  |
| *Global health status/QoL* | 72.6 (20.7) | 76.9 (19.7) | 4.3 (.4 − 8.2) | **.03** |
| Functional scales^1^ |  |  |  |  |
| *Physical functioning* | 82.3 (20.6) | 85.0 (18.5) | 2.7 (-.9 − 6.5) | .14 |
| *Role functioning* | 75.7 (29.8) | 78.1 (27.9) | 2.4 (-4.7 − 9.5) | .5 |
| *Emotional functioning* | 80.2 (19.5) | 87.4 (17) | 7.2 (3.8 − 10.5) | **<.001** |
| *Cognitive functioning* | 86.1 (16.7) | 88.4 (15.2) | 2.3 (-1.0 − 5.5) | .17 |
| *Social functioning* | 81.8 (25.3) | 86.7 (19.2) | 4.9 (.2 − 9.6) | **.042** |
| Symptom scales/items^1^ |  |  |  |  |
| *Fatigue* | 27.7 (27.9) | 24.6 (23.5) | -3.1 (-8.5 − 2.2) | .3 |
| *Nausea and vomiting* | 2.4 (8.5) | 3.0 (9.3) | 0.6 (-1.9 − 3.0) | .7 |
| *Pain* | 19.3 (25.7) | 15.4 (21.8) | -3.9 (-9.1 − 1.2) | .13 |
| *Dyspnea* | 12.4 (21.6) | 15.0 (23) | 2.6 (-2.3 − 7.5) | .3 |
| *Insomnia* | 31.5 (32.7) | 22.9 (26.9) | -8.6 (-13.9 − -3.4) | **.002** |
| *Appetite loss* | 11.6 (20.8) | 6.4 (17.3) | -5.2 (-9.3 − -1.2) | **.012** |
| *Constipation* | 9.4 (23) | 7.1 (17.8) | -2.3 (-7.3 − 2.8) | .4 |
| *Diarrhoea* | 8.2 (19.6) | 5.6 (16.1) | -2.6 (-6.5 − 1.2) | .18 |
| *Financial difficulties* | 1.5 (7) | 3.0 (13) | 1.5 (-.6 − 3.6) | .16 |

*SD* standard deviation*, ES* effect size*. Δ* difference between two values. *^1^* paired t-test.
