# Supplementary material for: Health-related Quality of Life in Localized and Metastatic Renal Cell Carcinoma: Insights from Patient-reported Outcome Measures
Source: Eur Urol Open Sci. 2026 Jan 21;84:50–7. doi: 10.1016/j.euros.2025.12.017 (PMC12859803; doi:10.1016/j.euros.2025.12.017)
Supplement: Supplementary Data 4 [file mmc4.docx]

**Supplementary Table 4**. QLQ-C30 scores for mRCC at T0 and T1.

|  | QLQ-C30 scores T0 (n=38), mean (SD) | QLQ-C30 scores T1 (n=38), mean (SD) | Δ QLQ-C30 scores (95% C.I.) | *p* |
| --- | --- | --- | --- | --- |
| Global health status/QoL^1^ |  |  |  |  |
| *Global health status/QoL* | 67.1 (27.7) | 75.4 (17.5) | 8.3 (.4 − 16.3) | **.040** |
| Functional scales^1^ |  |  |  |  |
| *Physical functioning* | 77.2 (19.4) | 82.5 (17.2) | 5.3 (-1.1 − 11.7) | .10 |
| *Role functioning* | 71.5 (30.7) | 76.8 (22.4) | 5.3 (-4.4 − 14.9) | .3 |
| *Emotional functioning* | 77.9 (18.7) | 84.7 (14.7) | 6.8 (-12.2 – 1.4) | **.015** |
| *Cognitive functioning* | 91.2 (13.3) | 93.9 (11.3) | 2.7 (-1.9 − 7.1) | .2 |
| *Social functioning* | 81.6 (25.1) | 88.2 (16.4) | 6.6 (-1.4 − 14.6) | .10 |
| Symptom scales/items^1^ |  |  |  |  |
| *Fatigue* | 31.6 (27.5) | 23.7 (19) | -7.9 (-15.5 − -.3) | **.042** |
| *Nausea and vomiting* | 3.1 (7.6) | 2.6 (6.2) | -0.5 (-3.1 − 2.3) | .7 |
| *Pain* | 18.4 (25.4) | 7.5 (12.7) | -10.9 (-19.8 − -2.1) | **.017** |
| *Dyspnea* | 21.1 (26.2) | 17.5 (24.2) | -3.6 (-11.1 − 4.0) | .4 |
| *Insomnia* | 23.7 (28.9) | 18.4 (26.5) | -5.3 (-13.1 − 2.6) | .18 |
| *Appetite loss* | 14.9 (24.1) | 7.9 (16.3) | -7 (-15.9 − 1.9) | .12 |
| *Constipation* | 9.7 (20.4) | 10.5 (20.7) | 0.8 (-8.8 – 10.6) | .9 |
| *Diarrhoea* | 4.4 (11.4) | 3.5 (12.9) | -0.9 (-6.3 – 4.5) | .7 |
| *Financial difficulties* | 5.3 (14.6) | 5.3 (19.8) | 0.0 (-5.1 – 5.1) | 1 |

*SD* standard deviation*, ES* effect size*. Δ* difference between two values. *^1^* paired t-test.
