# Supplementary material for: Health-related Quality of Life in Localized and Metastatic Renal Cell Carcinoma: Insights from Patient-reported Outcome Measures
Source: Eur Urol Open Sci. 2026 Jan 21;84:50–7. doi: 10.1016/j.euros.2025.12.017 (PMC12859803; doi:10.1016/j.euros.2025.12.017)
Supplement: Supplementary Data 5 [file mmc5.docx]

**Supplementary Table 5**. QLQ-C30 scores for M0 at T0 and T2.

|  | QLQ-C30 scores T0 (n=51), mean (SD) | QLQ-C30 scores T2 (n=51), mean (SD) | Δ QLQ-C30 scores (95% C.I.) | *p* |
| --- | --- | --- | --- | --- |
| Global health status/QoL^1^ |  |  |  |  |
| *Global health status/QoL* | 69.9 (21.7) | 79.7 (16.9) | 9.8 (4.9 − 14.7) | **<.001** |
| Functional scales^1^ |  |  |  |  |
| *Physical functioning* | 85.5 (18.3) | 89.9 (14.6) | 4.4 (-.7 − 9.5) | .086 |
| *Role functioning* | 72.9 (32.7) | 82.0 (22.8) | 9.1 (.7 − 17.6) | **.034** |
| *Emotional functioning* | 79.4 (20.4) | 86.6 (16.5) | 7.2 (2.2 − 12.2) | **.006** |
| *Cognitive functioning* | 86.6 (16) | 89.2 (15.2) | 2.6 (-1.7 – 7.0) | .2 |
| *Social functioning* | 79.7 (27.6) | 89.2 (17.9) | 9.5 (3.5 − 15.5) | **.003** |
| Symptom scales/items^1^ |  |  |  |  |
| *Fatigue* | 29.6 (31) | 20.5 (19.3) | -9.1 (-16.2 − -2.1) | **.012** |
| *Nausea and vomiting* | 2.3 (5.8) | 2.6 (6.1) | 0.3 (-1.7 − 2.3) | .7 |
| *Pain* | 19.9 (25.6) | 13.4 (20.6) | -6.5 (-13.3 − .2) | .058 |
| *Dyspnea* | 14.4 (23.3) | 11.1 (21.8) | -3.3 (-8.7 − 2.1) | .2 |
| *Insomnia* | 31.4 (33.6) | 20.3 (25.9) | -11.1 (-18.3 − -3.9) | **.003** |
| *Appetite loss* | 11.8 (21.9) | 5.2 (13.9) | -6.6 (-11.6 − -1.2) | **.017** |
| *Constipation* | 7.8 (21.7) | 7.8 (19.5) | 0.0 (-7.0 − 7.0) | 1 |
| *Diarrhoea* | 7.2 (18) | 4.6 (13.4) | -2.6 (-7.5 − 2.3) | .3 |
| *Financial difficulties* | 2.0 (7.9) | 0.0 (0) | -2.0 (-4.2 − .3) | .083 |

*SD* standard deviation*, ES* effect size*. Δ* difference between two values. *^1^* paired t-test.
