# Supplementary material for: Health-related Quality of Life in Localized and Metastatic Renal Cell Carcinoma: Insights from Patient-reported Outcome Measures
Source: Eur Urol Open Sci. 2026 Jan 21;84:50–7. doi: 10.1016/j.euros.2025.12.017 (PMC12859803; doi:10.1016/j.euros.2025.12.017)
Supplement: Supplementary Data 6 [file mmc6.docx]

**Supplementary Table 6**. QLQ-C30 scores for mRCC at T0 and T2.

|  | QLQ-C30 scores T0 (n=22), mean (SD) | QLQ-C30 scores T2 (n=22), mean (SD) | Δ QLQ-C30 scores (95% C.I.) | *p* |
| --- | --- | --- | --- | --- |
| Global health status/QoL^1^ |  |  |  |  |
| *Global health status/QoL* | 67.4 (23.4) | 74.2 (13.3) | 6.8 (-2.6 − 16.3) | .15 |
| Functional scales^1^ |  |  |  |  |
| *Physical functioning* | 78.5 (16.2) | 84.9 (13.5) | 6.4 (-1.6 − 14.3) | .11 |
| *Role functioning* | 69.7 (28) | 80.3 (18.3) | 10.6 (-3.6 − 24.8) | .13 |
| *Emotional functioning* | 74.2 (20.9) | 86.7 (11.7) | 12.5 (2.9 − 22.1) | **.013** |
| *Cognitive functioning* | 88.6 (15.8) | 91.7 (11.2) | 3.1 (-4.1 − 10.1) | .4 |
| *Social functioning* | 80.3 (21.6) | 90.9 (19.1) | 10.6 (-2.2 − 23.4) | .10 |
| Symptom scales/items^1^ |  |  |  |  |
| *Fatigue* | 31.3 (27.4) | 21.7 (14.3) | -9.6 (-22.4 − 3.2) | .14 |
| *Nausea and vomiting* | 1.5 (4.9) | 0.0 (0) | -1.5 (-3.7 − .7) | .16 |
| *Pain* | 18.9 (23.2) | 10.6 (18.2) | -8.3 (-19.0 − 2.3) | .12 |
| *Dyspnea* | 21.2 (28.3) | 19.7 (26.6) | -1.5 (-15.6 − 12.5) | .8 |
| *Insomnia* | 28.8 (29.6) | 15.2 (17) | -13.6 (-26.3 − -1.0) | **.036** |
| *Appetite loss* | 13.6 (22.2) | 4.6 (11.7) | -9.0 (-18.4 − .2) | .056 |
| *Constipation* | 10.6 (21.6) | 3.0 (9.8) | -7.6 (-18.4 − 3.5) | .17 |
| *Diarrhoea* | 4.6 (11.7) | 4.6 (15.6) | 0.0 (-6.5 − 6.5) | 1 |
| *Financial difficulties* | 6.1 (16.7) | 7.6 (17.6) | 1.5 (-4.0 − 7.0) | .6 |

*SD* standard deviation*, ES* effect size*. Δ* difference between two values. *^1^* paired t-test.
