# Supplementary material for: Health-related Quality of Life in Localized and Metastatic Renal Cell Carcinoma: Insights from Patient-reported Outcome Measures
Source: Eur Urol Open Sci. 2026 Jan 21;84:50–7. doi: 10.1016/j.euros.2025.12.017 (PMC12859803; doi:10.1016/j.euros.2025.12.017)
Supplement: Supplementary Data 7 [file mmc7.docx]

**Supplementary Table 7**. QLQ-C30 scores of the entire study population at T0 compared to the general Dutch population (25).

|  | QLQ-C30 scores study population at T0 (n=295), mean (SD) | QLQ-C30 scores for the Dutch general population (n=1000), mean (SD) (16) |
| --- | --- | --- |
| Global health status/QoL |  |  |
| *Global health status/QoL* | 69.6 (22.3) | 78.4 (19.1) |
| Functional scales |  |  |
| *Physical functioning* | 78.9 (20.5) | 89.7 (15.5) |
| *Role functioning* | 71.5 (30.4) | 88.5 (21.9) |
| *Emotional functioning* | 77.9 (20.4) | 84.2 (19.2) |
| *Cognitive functioning* | 87.2 (17.9) | 90.4 (16.1) |
| *Social functioning* | 81.4 (24.3) | 92.3 (19) |
| Symptom scales/items |  |  |
| *Fatigue* | 30.6 (27.3) | 22.3 (22.6) |
| *Nausea and vomiting* | 4.2 (11.1) | 2.9 (10.4) |
| *Pain* | 21 (26.6) | 17.9 (23.1) |
| *Dyspnea* | 15.4 (22.7) | 10 (20.4) |
| *Insomnia* | 24.4 (28.9) | 21.6 (26.2) |
| *Appetite loss* | 14 (21.6) | 4.9 (15.2) |
| *Constipation* | 9.7 (21) | 5 (13.6) |
| *Diarrhoea* | 6.3 (16) | 6.2 (16.9) |
| *Financial difficulties* | 4.3 (13.9) | 4.6 (15.8) |

*SD standard deviation*
