# Supplementary material for: Health-related Quality of Life in Localized and Metastatic Renal Cell Carcinoma: Insights from Patient-reported Outcome Measures
Source: Eur Urol Open Sci. 2026 Jan 21;84:50–7. doi: 10.1016/j.euros.2025.12.017 (PMC12859803; doi:10.1016/j.euros.2025.12.017)
Supplement: Supplementary Data 8 [file mmc8.docx]

**Supplementary table 8**. Baseline characteristics and QLQ-C30 scores of responders *vs* non-responders at T1 in patients with M0 and mRCC.

|  | Responders, n=127 (43%) | Non-responders, n=168 (57%) |
| --- | --- | --- |
| Age, mean (SD) | 66 (9.7) | 64.7 (11) |
| Gender, n (%) |  |  |
| Male | 92 (74) | 121 (71) |
| BMI, mean (SD) | 28 (11) | 27.8 (7.5) |
| Comorbidity, n (%)* |  |  |
| Heart disease | 22 (18) | 12 (15) |
| Stroke | 2 (1.6) | 3 (3.9) |
| Hypertension | 64 (52) | 33 (42) |
| Asthma, chronic bronchitis, COPD | 17 (14) | 10 (13) |
| Diabetes | 21 (17) | 12 (15) |
| Kidney disease | 62 (50) | 39 (50) |
| Liver disease | 6 (2.8) | 0 (0) |
| Anemia/other blood disease | 1 (0.80) | 11 (14) |
| Thyroid disease | 4 (3.2) | 0 (0) |
| Depression | 6 (2.8) | 3 (3.9) |
| Arthrosis | 2 (0.8) | 16 (21) |
| Backache | 45 (36) | 27 (35) |
| Rheumatism | 4 (3.2) | 1 (1.3) |
| Hereditary cancer syndrome | 1 (0.80) | 0 (0) |
| Unknown | 43 (20) | 34 (44) |
| Baseline QLQ-C30 scores, mean (SD)* |  |  |
| *Global health status/QoL* |  |  |
| Global health status/QoL | 70.7 (23.2) | 68 (20.1) |
| *Functional scales* |  |  |
| Physical functioning | 80.4 (20.4) | 79 (20.9) |
| Role functioning | 74.1 (30.2) | 71 (31.3) |
| Emotional functioning | 79.4 (19.4) | 77.5 (20.6) |
| Cognitive functioning | 87.6 (16) | 85.4 (19.7) |
| Social functioning | 81.3 (25.3) | 81.7 (22.5) |
| *Symptom scales/items* |  |  |
| Fatigue | 29.1 (28) | 31.5 (27.1) |
| Nausea and vomiting | 2.7 (8.3) | 4.8 (13) |
| Pain | 19.5 (25.7) | 22.3 (27.7) |
| Dyspnea | 15.3 (23.4) | 13.6 (21.3) |
| Insomnia | 29.6 (31.9) | 24.6 (30) |
| Appetite loss | 12.9 (22) | 14 (24.5) |
| Constipation | 9.7 (22.4) | 9.9 (21.1) |
| Diarrhoea | 7.3 (17.8) | 7.2 (18.3) |
| Financial difficulties | 2.7 (10.1) | 5.3 (17.1) |

**PRO*
